# Advancements of Medical Image Enhancement in Healthcare Applications

**DOI:** 10.1155/2018/7035264

**Published:** 2018-03-29

**Authors:** Shujun Fu, Ming Zhang, Chengpo Mu, Xiaohong Shen

**Affiliations:** ^1^School of Mathematics, Shandong University, Jinan, China; ^2^Tufts Medical Center, Tufts University, Boston, MA, USA; ^3^School of Mechatronical Engineering, Beijing Institute of Technology, Beijing, China; ^4^Department of Biomedical Engineering, Emory University, Atlanta, GA, USA

## 1. Introduction

As one of the world's largest and fastest-growing industries, healthcare engineering refers to all aspects of the prevention, diagnosis, treatment, and management of illness, as well as the preservation and improvement of physical and mental health and well-being through medical services [1, 2].

Medical imaging technologies play more and more important roles not only in the diagnosis and treatment of diseases but also in disease prevention, health checkup, major disease screening, health management, early diagnosis, disease severity evaluation, choice of treatment methods, treatment effect evaluation, and rehabilitation. The status of medical imaging technologies has increased continuously in healthcare applications [3–6].

Due to its ability to make the diagnosis and treatment of disease, image-guided surgery, and other medical links more timely, accurate, and efficient, medical image enhancement has become a routine task. Through producing excellent tissue uniformity, optimized contrast, edge enhancement, artifact elimination, intelligent noise reduction, and so forth, cutting-edge image enhancement helps doctors accurately interpret medical images, a crucial foundation for better diagnosis and treatment [3–6].

## 2. The Special Issue

Although the special issue focuses on medical image enhancement, other important image processing methods are reported from submitted papers, including image reconstruction, enhancement, segmentation, feature extraction, and recognition.

Next, we will introduce seven important works in the order of the above full-image processing procedure [5].

For the sparse reconstruction of X-ray computed tomography (CT) image with a low-dose projection data, W. Zhou and H. Xiang proposed a wavelet frame-based regularization method, where a novel balanced hybrid model was considered with two sparse regularization terms related to the latent solution and the frame coefficient. A fast alternative direction method was devised to solve the proposed model so that four subproblems can be easily solved. Numerical experiments verified the efficiency and the accuracy of the proposed model.

Efficient enhancement of noisy optical coherence tomography (OCT) images is a key task for interpreting them correctly in the ophthalmology for vision-related diseases. G. Liu et al. proposed a collaborative shock filtering to better enhance details and layered structures of human retina image. Noisy OCT image is first denoised by a 3D collaborative filtering method with a novel similarity measure; then, the denoised image is sharpened by a shock-type filtering for edge and detail enhancement. In order to improve image contrast for the detection of tiny lesions, a gamma transformation is also used to enhance the images within proper gray levels.

To better interpret medical images plays an important role in medical diagnosis and treatment. Y. Zhang and M.Q. An proposed a super-resolution reconstruction method based on transfer learning and deep learning, which contains one bicubic interpolation template layer and two convolutional layers. A SIFT feature-based transfer learning method is used to selectively add other types of images into the training dataset. The experiments on eight distinctive medical images showed the improvement of image quality with slightly sharper edges than other deep learning approaches in less time.

Image segmentation is a classic difficult problem linking low-level processing to high-level recognition. Three important works on three types of imaging modes are reported in this field. A fast and robust segmentation algorithm was presented by J. Marie et al. integrating the fuzzy clustering and the gravitational law. After the fuzzy enhancement of MRI images (T1/T2, FLAIR), the healthy structures in these images were separated from the unhealthy ones. Finally, the lesion contour is automatically outlined through an initialization-free level set evolution method. An 84%–93% overlap performance was obtained using clinical and synthetic brain datasets with different and heterogeneous types of lesions.

The segmentation of epicardial fat is also an important task for indicating the risk level for developing various cardiovascular diseases and predicting the progression of certain diseases. V. Zlokolica et al. proposed a semiautomatic approach for segmentation and quantification of epicardial fat from 3D CT images. After the user marks a patch corresponding to the epicardial fat, a 3D segmentation was done slice-by-slice integrating 2D processing using the fuzzy c-means clustering and ellipse fitting, followed by a filtering out of undesired parts of the target cluster.

Retinal layer thickness measurement offers important quantitative information for reliable diagnosis of retinal diseases and for the evaluation of disease development and medical treatment responses. This task critically depends on the accurate edge detection and segmentation of the retinal layers in OCT images. S. Luo et al. identified three most promising edge detection algorithms: Canny edge detector, the two-pass method, and the EdgeFlow technique. The quantitative evaluation results showed that the two-pass method outperforms consistently the other two methods in delineating and localizing the retinal layer boundaries in the OCT images. They also found that the OCT images contain more intensity gradient information than texture changes along the retinal layer boundaries.

With the development of digital X-ray imaging and processing methods, automatic categorization and analysis of massive digital radiographic images are urgently needed. N. Ren et al. developed a recognition method of radiographic positions using the frequency curve classification and gray information matching. Compared with predefined curve types (radiographic position) from a whole-body phantom image, a radiographic image is classified as one of the six positions with the help of image intensity similarity: the head, lung, lumber, pelvis, joint, and limbs. An average 93.78% accuracy is reported using the proposed method.
